# Ischemia/Reperfusion Injury: Pathophysiology, Current Clinical Management, and Potential Preventive Approaches

**DOI:** 10.1155/2020/8405370

**Published:** 2020-01-29

**Authors:** César Daniel Sánchez-Hernández, Lucero Aidé Torres-Alarcón, Ariadna González-Cortés, Alberto N. Peón

**Affiliations:** ^1^Sociedad Española de Beneficencia (SEB), Pachuca, Hgo, Mexico; ^2^Área Académica de Medicina, Universidad Autónoma del Estado de Hidalgo (UAEH), Mexico; ^3^Laboratorio de Microbiología, Escuela Superior de Apan (ESAp), UAEH, Apan, Hgo, Mexico

## Abstract

Myocardial ischemia reperfusion syndrome is a complex entity where many inflammatory mediators play different roles, both to enhance myocardial infarction-derived damage and to heal injury. In such a setting, the establishment of an effective therapy to treat this condition has been elusive, perhaps because the experimental treatments have been conceived to block just one of the many pathogenic pathways of the disease, or because they thwart the tissue-repairing phase of the syndrome. Either way, we think that a discussion about the pathophysiology of the disease and the mechanisms of action of some drugs may shed some clarity on the topic.

## 1. Introduction

Myocardial infarction (MI) or acute myocardial infarction is a term used to refer to an event of heart attack. MI occurs when the cardiac muscle is injured by hypoxia, which happens when a coronary artery is blocked [1]. MI is classified as being either an ST-segment elevation myocardial infarction (STEMI) or a non-ST-segment elevation myocardial infarction (NSTEMI). Moreover, unstable angina (UA) is closely related to NSTEMI, and together, these entities are referred to as non-ST-segment elevation acute coronary syndromes (NSTEACS). Both STEMI and NSTEACS share an underlying pathophysiology: a superimposed thrombus caused by a disruption of an atherosclerotic plaque, which results in subtotal occlusion (NSTEACS) or total occlusion (STEMI) of a coronary artery [2], thus causing damage at the heart's muscle through hypoxia induction.

The principal symptoms of MI are chest pain, which travels to the left arm or left side of the neck, shortness of breath, sweating, nausea, vomiting, abnormal heart beating, anxiety, and fatigue [3]. Risk factors include an advanced age, tobacco smoking, high blood pressure, diabetes, lack of physical activity, obesity, and chronic kidney disease [4]. Risk factors can be categorized into nonmodifiable and modifiable. Nonmodifiable risk factors include age of more than 45 years in men and more than 55 years in women, family history of early heart disease, and African-American race [5]. Modifiable risk factors include hypercholesterolemia, specifically related to elevation of low-density lipoprotein cholesterols (LDL-C), hypertension, tobacco abuse, diabetes mellitus, obesity, lack of physical activity, metabolic syndrome, and/or mental distress and depression [5]. The difference between both types of risk factors evidently lies in what can be prevented and what cannot.

There is an estimated five-million emergency department visits each year in the US for acute chest pain. Annually, over 800,000 people experience an MI, of which 27% die, mostly before reaching the hospital [6]. On the other hand, heart disease is Mexico's leading cause of death [7], accounting for 18.8% of total deaths, of which 59% are attributable to myocardial infarction.

In several studies, reperfusion therapy (fibrinolysis and coronary angioplasty) has demonstrated to produce a decrease in the morbidity and mortality associated with myocardial infarction [8]. However, the process of myocardial reperfusion can, paradoxically, enhance myocardial injury through inflammation, finally contributing to 50% of the final MI size [9]. The precise role inflammation plays in the setting of MI has been debated since the 1980s with the infiltration of leukocytes now being recognized as inflammatory mediators, as opposed to the previous concept of them being bystanders of the damage [10].

Nonetheless, in the therapeutic setting, the requirement for best preserving myocardial structure and function upon MI is to restore coronary blood flow as early as possible, using thrombolytic therapy and/or angioplasty [11], but as soon as blood flow is restored, an inflammatory response arises in the damaged section of the heart. This immune response further expands the damage made by the occlusion, originating a phenomenon known as myocardial ischemia reperfusion injury, or myocardial ischemia reperfusion syndrome (MIRS). Actually, MIRS is a major challenge to the treatment of MI [12], because its characteristic local and systemic inflammatory response is able to greatly enhance MI-derived damage, worsening the patient's prognosis [13]. Moreover, current pharmacopeia lacks a specific treatment for such condition. The treatment has been elusive because the immune-muscular-vascular interplay that characterizes MIRS is very complex, and a midpoint between downregulating the inflammatory tissue-damaging response and allowing the leucocyte-orchestrated reparative phase must be achieved.

On the other hand, ischemia reperfusion injury (IRI) is not exclusive to MI, as it also happens as a consequence to brain, kidney, liver, testis, or lung ischemia [14]. In such a tonic, we think that some lessons can be learned from these separate entities that may be applicable in the setting of MIRS. Also, information about MIRS-specific tissue-damaging and tissue-remodeling mediators is currently very vast, so that it may be useful to analyze the current baggage of knowledge on the topic, with aims to pinpoint some of the pathogenic pathways that may help to restrain MIRS upon blockage, as well as some strategies that may be of use for that purpose.

## 2. Pathophysiology of Myocardial Ischemia Reperfusion Syndrome

**Figure 1:**
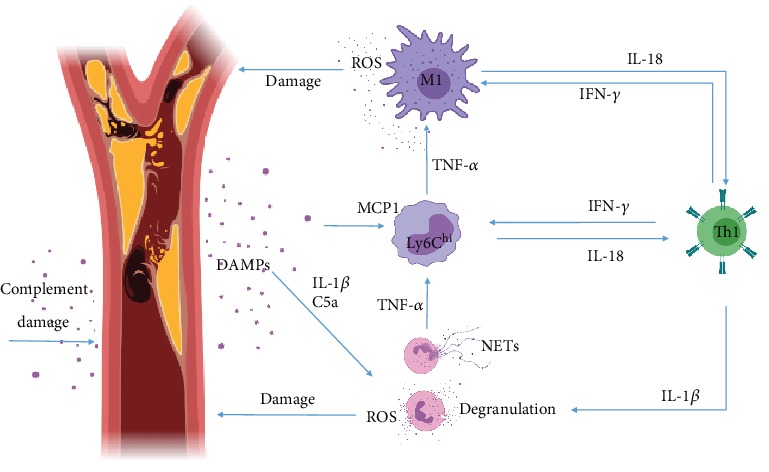
Inflammation during the Th1 tissue-damaging immune response of MIRS. Blood clots generate ischemia, which causes necrosis. Released DAMPs induce neutrophil and monocyte activation trough TLR and inflammasome activation, which in turn potentiate Th1 polarization. Inflammatory monocytes mature and become M1 macrophages. Tissue damage amplification comes in the form of NETs, granule components, and ROS produced by innate cells and direct complement attack.

**Figure 2:**
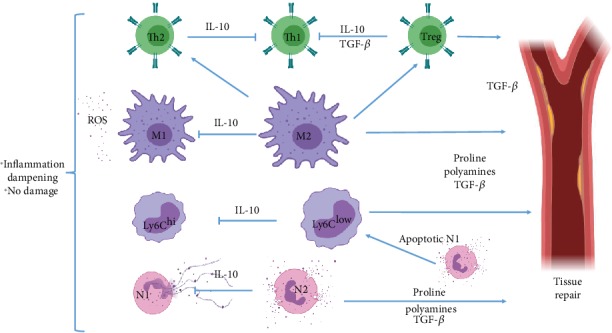
The Th2-mediated reparative phase of MIRS. N2 neutrophils and M2 macrophages both produce high levels of IL-10 to dampen N1, Ly6C^hi^, and M1-mediated degradation of tissue integrity. Also, M2 macrophages induce Th2 and Treg differentiation, while both suppress Th1 development, and Tregs thwart Th2 cells. M2 differentiation is possible by phagocytosis of the neutrophil apoptotic bodies. M2 and Treg cells mediate tissue repair.

In general terms, MIRS must be understood as a complex phenomenon that arises upon blood flow restoration, where reperfused leukocytes find many damage-associated molecular patterns (DAMPs), such as extracellular Ca^+^ and ATP released by necrotic cells, which induce the activation of many TLR pathways to promote an inflammatory response. Thus, an acute Th1 response is rapidly induced to clean the necrotic debris, but such an immune response, unfortunately, expands MI-associated damage [9, 15]. Myocardial reperfusion is unavoidable, as it occurs as a consequence to common MI treatments such as thrombolysis, angioplasty [16], and coronary bypass [17, 18]. At a later stage, the Th1-immune response subsides to a Th2-driven immunity, where leukocytes shift their phenotype in order to orchestrate tissue remodeling to avoid cardiac rupture [19]. A highly potent Th2 response, nonetheless, may induce pathological scarring, rendering the whole phenomenon as highly dependent on a very precise immune regulation. Thus, the mediators of this immunopathology must be precisely understood to find areas of opportunity for the development of a specific treatment (Figures 1 and 2).

### 2.1. Immunopathological Mechanisms of MIRS

The main trigger for MIRS is the vascular and cardiomyocyte cell death [11], which by the release of fragments of mitochondrial DNA, ATP, high mobility group box 1 protein (HMBGB1), and Ca^+^ into the extracellular space acts as DAMPs [20], inducing the activation of the NLRP3-inflammasome [21] and TLR9 [22], which converge on the activation of the myeloid differentiation primary response gene 88 (MyD88) and nuclear factor-*κ*B (NF-*κ*B) pathways, thus inducing the release of a number of inflammatory mediators, including monocyte-chemoattractant protein 1 (MCP1), interleukin-1*β* (IL-1*β*), IL-6, tumor-necrosis factor-*α* (TNF-*α*), and IL-18 [23]. Inflammasome activation amplifies IL-1*β* and IL-18 secretion by cardiac fibroblasts and induces the caspase-1-dependent death of nearby cardiomyocytes, termed pyroptosis—a highly inflammatory form of cell death, characterized by features that are typical of both apoptosis and necrosis [23].

Macrophage inflammatory protein-2*α* (MIP-2*α*), leukotriene B4 (LTB4), cytokine-induced neutrophil chemoattractant 1 (CINC-1), IL-8, CXCL8, and complement 5a massively recruit neutrophils [24] to infiltrate the MI-damaged area in the first few hours following onset of ischemia [25], peaking at days 1–3, and starting to decline at day 5. Neutrophils then generate high levels of reactive oxygen species (ROS), produce neutrophil-extracellular traps (NETs), and secrete granule components including myeloperoxidase and proteases, which exacerbate local vascular and tissue injury [26] with the purpose of removing necrotic cell debris from the affected zone [27] (Figure 1).

Along with neutrophils, complement proteins infiltrate the reperfused area. The complement is composed of 30 proteins and protein fragments, many of which are circulating as proenzymes and are activated by proteases in response to DAMPs. In this setting, all these proteins converge on two of the three common (terminal) complement pathways, which result in (a) inflammation to attract additional phagocytes (complements C3a, C4a, and C5a) and (b) activation of the cell-killing membrane attack complex (complement C5b-9 or MAC). Thus, the complement cascade amplifies MIRS-derived inflammation and damage [28] (Figure 1).

Both complement elements like C3a, C4a, and C5a and chemokines like MCP1 rapidly recruit monocytes [29] into the reperfused area. Such cells are produced in the bone marrow and are released into the blood in 2 waves, the first one being dominated by inflammatory Ly6C^hi^ monocytes (which peak at days 3–4 post MI) and the second one by anti-inflammatory Ly6C^low^ monocytes (which peak at day 7 post-MI). The infiltrating Ly6C^hi^ cells contribute to debris clearing and vascular/muscular damage, mainly through phagocytosis (for the earlier function) and ROS production (for the latter function) [30]. Monocytes then differentiate into M1-type macrophages, which have enhanced abilities to the phagocyte, produce ROS, and amplify inflammation through local antigen presentation and costimulation [31] (Figure 1). Subsequently, Ly6C^low^ monocytes start to infiltrate the reperfused area, and M1 macrophages start to differentiate into M2-type cells (which suppress T-cell activation through negative costimulation and IL-10 production and orchestrate tissue remodeling and vascularization by the secretion of TGF-*β*), to orchestrate tissue remodeling (as it will be discussed in the following section). Nonetheless, high levels of Th1-inducing factors deter the shift from an M1-type of macrophages to an M2 phenotype, thereby reducing the healing potential of the chronic MIRS phase [32] (Figure 1).

The systemic release of diverse cytokines and chemokines induces the activation of CD4^+^ T-cells, which in the acute phase of MIRS differentiate into a Th1 phenotype, releasing chemokines like CCL7 and cytokines like interferon-*γ* (IFN-*γ*), IL-2, and TNF-*α*, which as a cluster reinforce Th1 differentiation; enhance N1, Ly6C^hi^, and M1 cells' tissue-damaging abilities [33]; recruit CD8^+^ T-cells [34]; and enhance B cell activity [35]. Both CD8^+^ and B cells have been described to amplify inflammation during this stage and to produce damage on their own, by degranulation, in the case of T-cytotoxic cells [34], and antibody-mediated complement activation, in the case of B-lymphocytes [35] (Figure 1).

In this way, the immunopathology of MIRS can be succinctly described as the interplay between the innate and adaptive arms of the immune system, where a Th1-type immunity is critical for damage induction. In such a system, M1 macrophages and N1 neutrophils are key players on IRI induction, while the adaptive immunity component mainly amplifies the effector mechanisms of the aforementioned innate cells and complement.

### 2.2. The Th2-Mediated Reparative Phase of MIRS

On days 4-7 after MI, the Th1 tissue-destructive phase of MIRS enters a resolution stage, driven by a Th2 immune response induced by many changes in the cardiac microenvironment. This is regulated by the activation of endogenous inhibitory pathways that suppress the inflammatory phenotype in infiltrated leukocytes located in the MI zone [36].

After producing a high level of tissue damage, when most inflammatory debris have been cleared from the extracellular environment, neutrophils shift from their N1 phenotype to become N2-type cells. This change is accompanied by the production of high levels of IL-10, which aids in the suppression of the acute tissue-damaging Th1 response, by blocking the activation of CD4^+^, CD8^+^, B, N1, M1, and Ly6C^hi^ cells [37]. Moreover, they produce phosphatidylserine (PS), which facilitates ingestion of apoptotic neutrophils by macrophages, resulting in a phenotypic change for macrophages from the M1 to the M2 type, which secrete anti-inflammatory and profibrotic cytokines such as IL-10 and TGF-*β*, thus promoting tissue repair and vascularization, while aiding in the suppression of inflammation [38] (Figure 2).

Also, the polarization of monocytes and macrophages (M/M) from the tissue-damaging and proinflammatory Ly6C^hi^ and M1 phenotypes to the anti-inflammatory and tissue-repairing Ly6C^low^ and M2 phenotypes is critical to the reparative phase following MI [39], as these cells are able to produce an enzyme that is known as Arginase-1 (Arg-1). Such an enzyme catalyzes the conversion of L-arginine into L-ornithine, which is further metabolized into proline and polyamines. Both metabolites drive collagen synthesis and bioenergetic pathways that are critical for cell proliferation, respectively, thus contributing to tissue repair. Also, Arg-1 competes for the same substrate, but with more affinity, with the inducible-nitric oxide synthase (iNOS) enzyme, which is responsible for NO production [40]. In this way, M2 macrophages block ROS production by M1, N1, and Ly6C^hi^ cells, thus limiting the extent of tissue damage by the remaining N1 and M1 cells (Figure 2).

The shift in M/M and neutrophil phenotype is mirrored by CD4^+^ T-cells, as Th1 cells subside to a vaster Th2 population that apparently amplifies the strength of the reparative actions of the M/M population [41]. This effect may be due to Th2-derived high levels of IL-4 and IL-13, which are able to induce M2 activation in macrophages [34]. Moreover, recent studies suggest that invariant natural killer (iNK) T-cells and *γδ*T-cells have an important role in the settling of the Th1 acute inflammatory response through the secretion of anti-inflammatory cytokines such as TGF-*β* and IL-10, overall working with T-cells to dampen inflammation [42, 43]. Nonetheless, it has been observed that an enhanced Th2 response is able to induce pathological scarring with increased fibrosis in several settings [44, 45], in such a way that even the Th2 response must be controlled (Figure 2).

In the last decade, CD4^+^ CD25^+^ FoxP3^+^ T-regulatory (Tregs) cells have been recognized not only for their ability to dampen Th1 and Th2 lymphocyte activation and proliferation but also for their ability to downregulate innate immune cells' effector mechanisms [46, 47], while altering the cytokine milieu [48]. Tregs downregulate M1-macrophage activation and develop in parallel with M2 cells, presumably to control their level of activity [49]. In the setting of MIRS, Tregs have been shown to prevent cardiomyocyte apoptosis to limit further damage [48] and to downmodulate differentiation of fibroblasts into myofibroblasts, in order to avoid pathological scarring [50]. Their enhanced production of IL-10 has even been linked with a decrease in NKT cell activation [51]. In this way, they limit both the Th1- and Th2-mediated immunopathology [43] (Figure 2).

In a normal heart, there are a number of fibroblasts, which become activated during the reparative phase [52] mainly by the secretion of TGF-*β* [53], while the EDA-coated fibronectin produced by the newly transdifferentiated myofibroblasts induces extracellular matrix-protein (EMP) deposition [54]. Myofibroblast differentiation is also potentiated by the initial production of high levels of IL-1*β* and interferon-*γ*-inducible protein- (IP-) 10 [55], so that the extent of scarring is also determined by the significance of the Th1 response.

Moreover, activated myofibroblasts then modify the extracellular matrix environment, by the expression of EMPs like fibronectin and nonfibrillar collagens [55, 56], all of which support myofibroblast migration and adherence in order for them to close the wound.

On the other hand, from a wound-healing perspective, three phases of the process are recognized: (1) the inflammatory, (2) the proliferative, and (3) the remodeling stages, the first one being dominated by a Th1 response, the second one by Th2 immunity, and the third one being characterized by the reorganization, degradation, and resynthesis of the EM, in order to obtain maximum tensile strength. It is noteworthy that the latter process can last up to a year and only starts when Th2 cytokines have been downregulated, but also that in general, the strength and duration of each stage depends upon the strength and duration of the anterior phase [57]. In this way, Tregs have been linked to the transition from the Th1-mediated inflammatory stage to the Th2-mediated proliferative phase and finally to the remodeling phase, in such a way that these cells appear to promote the whole process of wound healing, while downregulating pathological scarring [58].

## 3. The Clinical Management of an MI Event

According to the European Society of Cardiology [59, 60], the best proceeding for the management of an MI is to obtain a 12-lead ECG as soon as possible, with the optimum proposed time lapse of 10 minutes in order to determine the precise location, extension, and kind of myocardial infarction for each patient, in order to personalize the surgical procedure. Pain relief should be practiced as soon as possible to avoid the increase of the heart's workload. It is usually done with the use of titrated opioids, although it is currently under debate if such drugs may interfere with the action of antiplatelet aggregation agents [61, 62]. Oxygen should also be administered in patients whose O_2_ saturation is less than 90%, along with a mild tranquilizer in order to reduce stress. When the diagnosis of STEMI is made in a prehospital setting, immediate activation of the catheterization laboratory is encouraged, in order to reduce treatment delays and patient mortality [63]. Either way, after diagnosis, pain management, and oxygenation, the next step is an attempt to lyse the blood clot by the use of thrombolytic drugs [59, 60].

Two scenarios may happen after thrombolysis: (1) the heart may recover blood flow or (2) the heart's blood flow alterations may persist. In the first case, MIRS starts upon thrombolysis, while in the second, primary percutaneous coronary intervention (PCI) is the preferred strategy that should be applied to patients with confirmed STEMI diagnosis within the first 12 h of symptom onset. In this second scenario, MIRS will happen after surgical reperfusion.

### 3.1. Periprocedural Pharmacotherapy

Patients undergoing primary PCI should receive aspirin and a P2Y_12_ inhibitor, in order to dampen platelet aggregation. The oral dose of aspirin should be administered without an enteric coat to ensure rapid action [59, 60].

Routine postprocedural anticoagulant therapy is not indicated after primary PCI, except when there is a separate indication for either full-dose anticoagulation or prophylactic doses for the prevention of venous thromboembolism in patients requiring prolonged bed rest, but ECG monitoring for arrhythmias and ST-segment deviations is recommended for at least 24 h after symptom onset in all STEMI patients. Afterwards, lifestyle changes are suggested to patients in order to prevent further risks [59, 60].

It should be noted that current medical guidelines do not mention any anti-inflammatory treatment to cope with MIRS, in such a way that the phenomenon still allows for an enhanced risk of post-MI injury progression [9].

## 4. Immunoregulation as a Modern Alternative to Immunosuppression

While the pathophysiological mechanisms of MIRS have been extensively studied, to the point where many inflammatory mediators, such as leukocytes and cytokines, and their role in the whole phenomenon are known, current pharmacopeia lacks a specific treatment to avoid MIRS. Despite this, much research has been done to attack the different pathways involved in postischemic injury progression, and it may be important to review these attempts in order to understand what has failed and what could be done.

As stated in the above sections, neutrophils have been identified as major targets in MIRS because of their ability to massively infiltrate the infarct area upon reperfusion [64], to locally produce high levels of tissue-damaging ROS, NETs [65], and granule components such as myeloperoxidase and proteases. As such, research using animal models has shown that the inhibition of their tissue-damaging mechanisms [66] and recruitment into the reperfusion site [67] may be a viable option to limit MIRS-associated damage. Nonetheless, clinical trials using *α*CD11/CD18 integrin blocking antibodies to avoid neutrophil recruitment during myocardial reperfusion have shown limited success in the reduction of MI size and the improvement of short-term (30 days after infarct) clinical outcome [68, 69].

**Figure 3:**
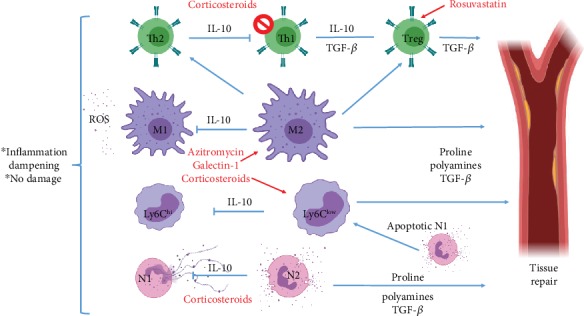
Immune-regulatory drugs could thwart destructive inflammation and promote tissue repair. Corticosteroids could enhance M2 differentiation while blocking NET, ROS, and granule-component deposition, thus blocking inflammatory damage. Also, azithromycin and rosuvastatin may induce cardioprotective leukocytes.

Despite the inflammatory, tissue-damaging role that neutrophils have on the acute phase of MIRS, after ≈7 days, the inflammatory Ly6G^+^ CD206^−^ neutrophil population is replaced by a Ly6G^+^ CD206^+^ population that has been described to play an important role in the orchestration of the reparative phase, as reviewed in [70]. Also, apoptotic neutrophils induce an M2 phenotype in infiltrated macrophages upon their phagocytosis, which inhibits the macrophage proinflammatory tissue-damaging response and leads them to produce IL-10 and TGF-*β* [71, 72]. Importantly, IL-10 may serve to dampen both Th1 and Th2 inflammation, thus inhibiting MIRS-derived damage, as well as excessive tissue scarring during the reparative phase, while TGF-*β* may also play an important role in infarct revascularization (Figure 3).

Thus, blocking neutrophil recruitment may not be a good alternative to reduce reperfusion-derived damage. Rather, the inhibition of the pathogenic effects of such cells may have a beneficial effect on MIRS. For instance, glucocorticoids have been shown to inhibit NET formation [73] and ROS production [74], while enhancing neutrophil mobilization [75], which renders them as good candidates for the reduction of neutrophil-derived damage (Figure 3).

Moreover, upon activation and apoptosis, neutrophils release proinflammatory alarmins that recruit inflammatory Ly6C^hi^ monocytes [76], which are also important players in the acute production of ROS. In later stages (1-2 days after MI), these cells undergo differentiation (peaking at 3-4 days post MI) into the proinflammatory tissue-damaging M1-type of macrophages [77]. Also, M1 macrophages can be directly recruited and activated through MCP1 early production by damaged endothelial cells and cardiomyocytes [78, 79]. Either way, increased Ly6C^hi^ cell counts after reperfusion have been associated with increased MIRS-derived damage [80, 81] as well as M1 macrophages, which further potentiate IRI [82]. At day 7 post MI, both the Ly6C^hi^ and the M1-macrophage populations subside to the inflammation-resolving tissue-remodeling Ly6C^low^ monocytes and M2 macrophages, which by Arg-1 expression deplete NO production and produce IL-10, TGF-*β*, polyamines, and proline, thus undermining the inflammatory tissue-damaging acute phase of the MIRS and promoting tissue repair and vascularization. Nonetheless, an excess of both M2 macrophages and Ly6C^low^ monocytes has also been associated with pathologic myocardial scarring [19, 83]. In this way, both the proinflammatory and the anti-inflammatory M/M fractions can have a pathogenic role in MIRS, so that they represent an important target to limit MIRS-associated damage as a whole (Figure 3).

Despite these evidences, blocking the inflammatory M/M recruitment into the MI zone might not be beneficial, as the adoptive transfer of M2 macrophages and Ly6C^low^ monocytes has shown to reduce MIRS-associated damage [84–86], so that the avoidance of M/M recruitment in the first place may limit the reparative phase of MIRS. On the other hand, M/M phenotype modulation to dampen such a cell's ability to produce oxidative and inflammatory stress may be a better strategy. Following this line of thought, IL-1*β*-blocking antibodies have been proposed as therapeutic alternatives to limit IRI, but results obtained from clinical trials have been contradictory, ranging from promising to discouraging [87–89]. Disregarding the results from clinical trials, animal models of this disease have shown a good limitation of MIRS-associated damage in relation to the use of IL-1*β*-blocking antibodies administered to diabetic rats, which has more translational value because most MI patients are diabetic. Importantly, the MIRS blockage with this kind of antibody was effective to improve systolic function even when it was administered 80 days after reperfusion [90, 91] (Table 1).

Current data on the phenomenon does not allow an exact explanation of this phenomenon, but it can be speculated that the lack of effect in some cases may be due to a vast array of M1-inducing cytokines and Ly6C^hi^-recruiting chemokines, other than IL-1*β*, being secreted at the MI zone upon reperfusion. Several cytokines and chemokines produced during MIRS, like TNF-*α*, IFN-*γ*, and MCP1, are known to have concomitant effects on the activation of inflammatory pathways like NF-*κ*B [92, 93], PI3K/Akt [94], and JAK/STAT [95] in such a way that the inhibition of just one of the cytokines that signal through any of those pathways would not be able to have a consistent effect on the reduction of MIRS-associated damage (Figure 3).

In such line of thought, chemerin-15 [85] and netrin-1 [86] have been used in animal models to induce an M2 phenotype in macrophages during ischemia reperfusion, with the effect of reducing lesion size. Concordantly, glucocorticoid administration has shown to induce an alternative activation in macrophages, in such a way that they protect against inflammatory injury and are able to induce Treg expansion [96]. Such an effect may be attributable to the inhibition of the NF-*κ*B pathway. Also, widely available drugs like azithromycin have shown to induce M2-type activation in macrophages to protect from ischemic stroke injury [97], in an effect associated with the inhibition of the PI3K/Akt pathway [98]. Moreover, the modulation of innate immunity using the C-type lectin, galectin-1, has also been proven to effectively dampen inflammation, mainly through AAM induction [99]. Interestingly, galectin-1 knockout mice showed enhanced cardiac inflammation (characterized by increased numbers of macrophages, natural killer cells, and T-cells) and a reduced frequency of regulatory T-cells that are associated with impaired cardiac function and ventricular remodeling. In the same study, the authors treated infarcted mice with recombinant galectin-1, which led to attenuated cardiac damage [100] (Table 1).

Whether this strategy induces pathological scarring was not evaluated, but the possibility should not be ruled out. To our notice, no clinical trials have been made exploring any immunoregulatory drug that has a direct effect on the M/M phenotype, and it may be important to gather such data due to a wide variation between the characteristics of MI in animal models and the clinical reality in human patients [19] (Figure 3).

On the other hand, CD4^+^ T lymphocytes and B cells are recruited within the first 90 minutes after reperfusion and appear to play a pathogenic role during the acute stage of MIRS, presumably because of their ability to promote an inflammatory tissue-damaging phenotype in M/M cells [33, 101]. Furthermore, in the tissue-remodeling stage of MIRS CD4^+^, T-cells may also play a pathological role, as they have been described to induce excessive scarring [102]. Nonetheless, there is an increasingly clear role for Treg cells in the dampening of both the pathogenic Th1 and Th2 inflammation phenomena [103] that is supported by several data (Figure 3).

Firstly, the induction of IL-10 secreting Treg cells by intranasal troponin administration shortly after reperfusion has shown to reduce MIRS-associated damage by 50%, evaluated 1.5 months after reperfusion [104]. Moreover, pharmacologic activation/recruitment of CD4^+^ CD25^+^ FoxP3^+^ cells using a super-antagonistic *α*CD28 antibody has been linked to a change in the phenotype of macrophages from M1 to M2, which promotes an enhanced, but not pathogenic, healing through the local production of TGF-*β* [49]. The observed suppression of pathogenic scarring may be due to a direct effect for Treg cells in the modulation of a fibroblast phenotype, in such a way that the latter cells migrate less, thus limiting their ability to form bigger scars [50] (Figure 3) (Table 1).

Another potentially important strategy to limit MIRS may be the use of statins, as they have been rendered as potent cardioprotectors that have an interesting effect on T-cell activation [105]. For instance, rosuvastatin has been shown to limit MIRS through Treg expansion in a murine model [106], but the effect may not be exclusive to animal models, as a meta-analysis performed by Sorathia et al. shows a vast increase in Tregs in patients that use rosuvastatin [107] (Figure 3) (Table 1).

Another important early player in the field of MIRS is the complement cascade, where C1 and C5/C5a proteins have been targeted. While C5 has been targeted with limited success on limiting IRI size [108, 109], C1 inhibition with monoclonal antibodies was able to reduce injury on several clinical trials [110–112], so that complement-blocking antibodies, like Cetor or Berinert, may be used concomitantly to reduce IRI extension. Additionally, corticosteroids have been used to regulate complement-gene expression and activation [113, 114] (Figure 3) (Table 1).

Finally, the potentiation of cardiomyocyte survival should be considered as a valuable alternative to be coopted in the treatment of MIRS. An interesting approach is the modulation of the low-density lipoprotein receptor-related protein 1 (LRP1), which is able to both downregulate the NF-*κ*B-related inflammation during MIRS and enhance cardiomyocyte survival through the activation of the PI3K/Akt and ERK1/2 pathways in such cells, as thoroughly reviewed in [115]. As an example of this approach, a clinical trial using plasma-derived alpha-1 antitrypsin, an agonist of the LRP1 receptor, showed shorter time-to-peak creatine kinase myocardial band (CK-MB) values [116] in relation to a significant reduction on CRP [117] (Table 1).

### 4.1. As Paracelsus Said: The Dose Makes the Potion

In the 70s, a word of caution was emitted against the use of corticosteroids to treat MIRS as it was observed that in some studies, it caused myocardial thinning and delayed healing [119–121]. Nonetheless, in all these studies, high doses of such hormones were administered and for prolonged times. In this way, even a decade later, this dosing was questioned by studies comparing MI size and healing pace between high and low corticosteroid dose groups [118], finding that as Paracelsus said, “the dose makes the potion.”

It can be speculated that the high doses used in such studies blocked the proliferative and remodeling stages of MIRS, along with the inflammatory phase that was initially the intended target. Nowadays, a protective role for corticosteroids in MIRS has been described in both experimental [122, 123] and clinical settings. Concordantly, a meta-analysis by Giugliano et al. [124] showed this cardioprotective effect for corticosteroids in MIRS in patients. On the other hand, corticosteroids have been successfully used to reduce IRI in kidneys [125], liver [126], and brain [127], with the added benefit of attenuating pathogenic fibrosis during the reparative phase [128].

In this way, the current understanding on the pathophysiology of MIRS and a brief review about the use of such drugs in MIRS-reduction allow us to think that a low dose of corticosteroids administered prior to reperfusion may help to reduce the inflammatory damage of such a syndrome, while allowing the healing phases of the syndrome.

## 5. Conclusions

MIRS is an unavoidable consequence of MI, with the potential to duplicate the damage made by the ischemic condition. As such, it represents a serious complication to one of the most prevalent diseases worldwide. Designing an effective therapy for such a condition has been challenging because the inflammatory phenomenon behind its pathophysiology is very complex. First, it involves a Th1 response that greatly contributes to tissue damage, which is relatively easy to dampen, but a chronic Th2-type immune response that contributes to the resolution of the inflammatory damage, and tissue remodeling comes later, and its suppression has been associated with increased damage.

As such, a therapy that downregulates the acute Th1 tissue-damaging response, but promotes the later Th2 tissue-repairing phase of the disease, appears to be a good choice. Some well-known, widely used drugs, like rosuvastatin, azithromycin, corticosteroids, Cetor, or Berinert, have been purported as candidates to treat MIRS in the experimental setting, producing good results. Nonetheless, much research is needed in order to confirm such findings as they have not been used concomitantly, and a correct dose may be challenging to find, as too much Th1 undermining may result in a weak reparative stage, but too little may not properly limit the damage.

Innate immune cells, like M/M and neutrophils, appear to be good targets, because they are effector mediators of the damage and because they can regulate the adaptive immune response, both in potency and in profile, so that drugs like azithromycin, which can induce an M2 phenotype in macrophages, or corticosteroids that can reduce ROS production in both cell types could have a positive effect on MIRS management. Also, rosuvastatin may be cardioprotective beyond its effects on dyslipidemia, as it can recruit Treg cells at the injured heart. Such lymphocyte population has been associated to the resolution of both the Th1- and Th2-type responses, thus allowing a healthy scar maturation.

Another point to be considered is the rational use for corticosteroids, as they can limit the extent of MIRI and induce protective leukocyte populations, but overdoses with such a drug have produced myocardial thinning and delayed healing.

Finally, complement-blocking antibodies have been used successfully in the clinical setting, so that they may be coopted with the aforementioned drugs to design a more complete treatment.

## Figures and Tables

**Table 1:** Main perspectives for the treatment of MIRS.

Clinical trial or animal model	Treatment	Proposed mechanisms of action	Findings	Reference
CT	*α*CD11/CD18	Reduction of neutrophil recruitment	No difference in baseline, angiographic of angioplasty characteristics	[68]
CT	*α*CD18	Reduction of neutrophil recruitment	No differences in coronary blood flow, infarct size, or ECG ST-segment elevation resolution	[69]
AM	Chemerin-15	Enhanced AAMs and IL-10; reduced ROS, neutrophils, IL-6, and TNF-*α*	Amelioration of MI	[85]
AM	Netrin-1	Reduction of neutrophil and macrophage recruitment, induction of AAMs	Decreased cardiomyocyte apoptosis	[86]
CT	*α*IL-1*β*	Reduction of M/M inflammatory activation	Enhanced hemodynamics and left ventricular remodeling	[87]
CT	*α*IL-1*β*	Reduction of CRP	No differences with the placebo-treated group	[88]
AM	*α*IL-1*β*	n.a.	Reduces infarct size and improves left ventricle remodeling	[90]
AM	Azithromycin	Induction of AAMs, inhibition of the PI3K/Akt pathway	Neuroprotection on an animal model of stroke	[97]
AM	Rosuvastatin	Treg expansion, reduction of inflammatory infiltrates	Reduced cardiac troponin I, infarct size	[106]
CT	*α*C5	n.a.	No reduction of infarct size but improved survival	[108]
CT	*α*C5	n.a.	Enhanced survival	[109]
CT	*α*C5	n.a.	Reduction of troponin T and creatine kinase-MB	[110]
CT	C1-esterase inhibitor	C1, C3c, and C4 reduction	No difference in postoperative complications, hospital stay, or in-hospital mortality	[111]
CT	C1-esterase inhibitor	C3a and C4a reduction	Enhanced mean arterial pressure, cardiac index, and stroke volume. Lower levels of cardiac troponin	[112]
AM	Low dose of methylprednisolone	n.a.	Reduced infarction size and scar	[118]
CT	Alpha-1 antitrypsin (AAT)	Reduction of CRP	Lower creatine kinase myocardial levels	[116]
AM	Galectin-1	Reduction of macrophages, NK cells, and T lymphocytes. Increase in Tregs	Enhanced heart's contractility	[100]
AM	Intranasal troponin	Increased IL-10 and reduced IFN-*γ*	Reduction of infarct size	[104]
AM	Super-antagonistic *α*CD28 antibody	Treg and AAM induction	Increased collagen de novo expression, decreased rates of left ventricular ruptures	[49]

Abbreviations: CT: clinical trial; AM: animal model; *α*CD11/18: anticluster of differentiation 11/18; *α*C5: anti-C5 complement protein; AAM: alternatively activated macrophages; ROS: reactive oxygen species; M/M: monocyte/macrophage; CRP: C-reactive protein; Treg: T-regulatory cell, IL-1*β*: interleukin-1*β*; IL-10: interleukin-10, ECG: electrocardiogram; n.a.: not available; MI: myocardial infarction.
